# A Manual of Midwifery

**Published:** 1832-07-01

**Authors:** 


A MANUAL OF MIDWIFERY, OR COMPENDIUM OF GYNÆCOLOGY AND PAIDONOSOLOGY, &c. By *Michael Ryan*, M. D., &c. Small Octavo, pp. 737. Third edition, with plates, 1831. Price 12s.

THE talented and industrious author of this manual has rendered it totally impossible for us to analyze the work. It is itself a condensed analysis of most other works on the subject. Let himself speak.

"It has been long remarked that all our knowledge of any science might be easily compressed into a small compass, by confining it to a simple enunciation of fact and inference. It appeared to me that this principle might be applied with peculiar advantage to the Science and Practice of Obstetricy; and it was by a rigorous adherence to it, that I have accomplished this work. My object was to concentrate facts and opinions, to describe them in language as simple as possible, under such arrangement and classification as would afford the easiest reference to the immediate object of research." *Preface.*

Dr. R. alludes to the inconvenience which he experienced in the earlier years of his practice for want of such a compendium; and states that, from the flattering encouragement the two first editions have received in this country, on the Continent, and in America, he has been stimulated to spare no pains in preparing this impression with all the improvements of which it was susceptible. Every line of this third edition has been re-written, and carefully revised, referring, on all occasions, to the last editions of the standard works on midwifery.

"In publishing this compendium, my object has been to present students and young practitioners with a concise, yet comprehensive, view of the exact state of Obstetric knowledge, by compressing into a small space all that is essential to be known upon the subject." ix.

Dr. Ryan has proposed a new nomenclature, which we hardly expect to see soon adopted, in consequence of the general disinclination to learn new names, of old things. We must give Dr. Ryan's nomenclature, however, the praise of being more classical and erudite than that which is in general use among obstetric writers. Whether it is entirely original, we have not time or inclination to inquire; but we take it for granted that it is, from Dr. Ryan's own assertion. Embryotomy, dystocia, and one or two other terms are, of course, adopted from preceding systems of terminology. Dr. R. claims the merit of being the first to change man-midwife into the more erudite name of obstetrician—and midwifery into obstetricy. Velpeau and others have adopted this improvement.

"The work is divided into four Parts, and each is subdivided into Articles and Sections.

The *First Chapter* is entitled Gynæcotomy, and comprises the Anatomy of the Sexual Organs of Women.

The *Second Chapter* is headed Gynæcophysiology, and is divided into four Articles; 1. Nubility; 2. Utero-gestation, or Pregnancy; 3. Delivery; and 4. Lactation, or the Period of Suckling.

The *Third Chapter* is entitled Gynæcopathology, and is divided into four Articles; 1. Parthenosology, or diseases of nubility, or of women in the unimpregnated state; 2. Encyonosology, or diseases of utero-gestation or pregnancy; 3. Dystocia, difficult labour, or morbid parturition; 4. Lochionosology, or diseases of puerperal women.

The *Fourth Chapter* is designated Paidonosology, or diseases of infants and children." xiv.

As we have before observed, it is quite impossible to even attempt an analysis of such a work as this, displaying, as it does, the laborious research of years, and a solidity of condensation and concentration which we could not hope to equal, much less to surpass. All we can do is to strongly recommend a work which indeed has proved its own merits by arriving at a third edition, and to offer an ample specimen of the performance, taken at random, and by no means the most favourable sample which we could adduce.

"Puerperal Fever—Fatal Child-bed Fever.

It is become fashionable of late years to question the opinions of our predecessors, and in no instance more remarkably so than on the nature of the disease under consideration. There is no disease about which there is so much dissonance of sentiment as puerperal fever; many of the most eminent obstetricians in Europe and America entertain discordant opinions as to the pathology of the disease. Some contend that ' it is excessively absurd to speak of puerperal fever, except as a symptom of a local disease. (Conquest, Blundell, Duges,) while others, as Hamilton, Burns, Joseph and John Clarke, Roux, Velpeau, Mastoscheck, Raimann, Hartman, Boer, Schiffner, Festi, Belletzky, and Editors of the Edinburgh Medical and Surgical Journal, 1824, entertain a different opinion.

The first accurate account of puerperal fever appeared in the Mem. de l'Acad. Roy. de Soc. 1746, for a full detail of which I refer the reader to my Essay in the London Med. and Surg. Journ. 1829, vol. iii. p. 18.

Dr. Gooch has given an imperfect history of this disease, and absolutely contradicts himself. At one time he strenuously maintains that it is peritonitis, or as he unfortunately designated it, peritoneal fever; and in future pages he tells us, that depletion is not the remedy in some cases; so that he is and is not a monopathologist. Subsequent experience has proved, beyond all doubt, that the disease under notice is not peritonitis. This will appear by a reference to the essays of Dr. Conquest and M. Tonnellé, which I have inserted in full in the London Med. and Surg. Journ. 1830, vol. v. No. 25.

Dr. Conquest objects to the vague term puerperal fever, under which are confounded the most opposite diseases of the brain, chest, abdomen, and pelvis. He informs us, that in many cases the morbid appearances are not sufficient to account for death. He has only found a fallopian tube, or ovary inflamed. In other cases there are hysteritis, uterine phlebitis, gangrene of the uterus, and agglutination of all the pelvic viscera. The type of the inflammation will be so modified by circumstances as scarcely to be recognised as the same disease in different women, in different districts, and during peculiar constitutions of the atmosphere. He thinks the disease communicable by medical men and nurses, that gestation and parturition produce a change in the physical condition of the female, which so modifies disease as to give it a specific character. This is decidedly the general opinion of medical practitioners. He is certain that the disease may commence during gestation, from mental depression, impure air, bodily fatigue, low living, improper food, &c. It may commence as hysteritis, or peritonitis. The approach of the disease is often so obscure as to elude detection.

When the disease is inflammatory, copious depletion is the remedy: when epidemic, it defies all treatment. The greatest caution must be observed in using the lancet. Leeches and cupping are often valuable adjuvants; hot turpentine applied to the abdomen is of great use, it should be used with flannel every six hours for ten minutes at each time, until high erythematous efflorescence takes place. In my own practice I use it freely at once, until the erythema appears. Oil of turpentine alone, or combined with castor-oil and laudanum, is a valuable purgative in all cases, not admitting of much reduction of power. Opium and mercurials, in large doses, are invaluable after bleeding and purging. The reader will find further observations on the efficacy of these remedies on referring to the article Peritonitis in a former page. Camphor in scruple doses, combined with opium, is a valuable anodyne in cases of great restlessness. Digitalis, nitrate of potass, ipecacuanha, and antimony are valuable adjuvants, but must not be relied on exclusively. Such are the leading facts detailed by Dr. Conquest in his Observations on Puerperal Inflammation, commonly called Puerperal Fever, read before the Hunterian Society of London, February 1830. M. Tonnellé prefers the term puerperal fever to peritonitis or metro-peritonitis, because it is more comprehensive than the others; it expresses nothing by itself, and does not prejudice the nature of the disease. He informs us, that the disease raged at the Maternité during the year 1829, with more violence than at any period since the establishment of that hospital. He gives the following account of the Pathology. 'It is in the most violent cases that the integrity of the peritoneum is most constantly preserved; it is mostly inflamed in the hypogastric region. The internal surface of the uterus is almost always covered with a putrilaginous matter of a red brown colour, and of an insupportable fetidity. The proper tissue of the uterus is rarely changed, except by ramollissement or putrescence. Suppuration of the veins and lymphatics of the uterus, is seen in three out of five cases of puerperal fever, and is nearly as constant as peritonitis. It may extend to the ovarian, hypogastric, and abdominal veins. This phlebitis exists generally on the sides of the uterus. M. Dance held that it existed more frequently near the insertion of the placenta. The lymphatics may take up fetid fluids after delivery, and become inflamed. *The presence of pus in the vessels, and its necessary transmission through the circulation, causes rapidly a palpable infection of the blood, and a certain number of phenomena which impress on puerperal fever an especial character, a characteristic physiognomy.* In two hundred and twenty-two cases, the uterus was affected in one hundred and ninety-seven, and the peritoneum in one hundred and ninety-three; there was pus in the uterine veins, and lymphatics in one hundred and thirty-four cases. The terms peritonitis or metro-peritonitis cannot be applied to many of the alterations described, but the term puerperal fever may be applied to all—it prejudices none of them.' (Des Fièvres Puerperales observées à la Maternité de Paris, pendant l'Année 1829, &c. Par M. Tonnellé. Arch. Gen. de Med. Mars et Avril, 1830; Lond. Med. and Surg. Journ., July 1830.)

### Symptoms of Puerperal Fever.

The disease commences from one to eight days after delivery, the patient complains loudly of the severity of the after-pains, and refers all her suffering to the region of the pubis and uterus. The disease is ushered in with much shivering or rigors, the pulse varying from 120 to 160. There is great prostration of mind and body, and a sense of undescribable oppression about the præcordia, and often an utter carelessness about the infant. There is always intense pain in the head. Sometimes the skin is hot and dry, face flushed, and indicative of great distress of mind. Respiration anxious and profound, as if performed with a sigh. Pain in the uterine region aggravated by pressure (metro-peritonitis,) which is first confined, but soon extends to the whole abdomen. There is much restlessness and jactitation, the abdomen rapidly becomes distended as large as before delivery, the pain sometimes diminishes or ceases. Nausea, or vomiting, at first of bilious, and at length of a coffee-coloured matter occurs ; sometimes there is diarrhœa or constipation, the face is peculiarly ghastly, and becomes pale, circle about the eyes livid, lips dry, headache, tinnitus aurium, tongue pallid or red, secretion of milk suppressed, lochia continued, but may be suppressed, according to Dr. John Clarke ; urine sparing and high coloured, bowels confined. The eyes become dull, the pupils dilated ; the nose sharpened, the cheeks hectic or pale, the lips purple, or the face livid, as in the last stage of typhus ; the forehead and chest are covered with cold perspiration. The patient lies on her back, with the lower limbs drawn up, to relieve her respiration, which is much impeded by the tumefaction of the abdomen from gas or effusion ; the tongue becomes black, mouth aphthous, teeth covered with sordes, the breath cadaverous, vomiting of a brown pitchy fetid matter, involuntary alvine evacuations of black colour, with fetid odour ; the abdomen becomes less swelled, the countenance is inanimate, the alæ nasi are tremulous, the respiration becoming more and more panting. The patient speaks incoherently, mutters to herself, or is delirious and attempts to get out of bed. The pulse is so rapid as not to be reckoned, the perspiration is cold and clammy ; there is subsultus tendinum, and death soon closes the scene. Dr. W. Hunter stated in his Lectures, 'treat the disease as you will, three out of four will die.'

The disease attacks every temperament and constitution, the strong and weak, the young and middle aged, the poor and rich ; but is more common among the poor and middle classes, and from the age of puberty to thirty-five, than at any other period. It occurs most frequently within the first eight days after delivery. It is one of the most fatal diseases. Lowder considered the inflammation of the peritoneum was erysipelatous, and the fever typhoid. He said that all patients who were bled, died ; and that two were cured by taking a gallon of decoction of bark daily. It is most rapid in its fatal career, often terminating in forty-eight or thirty-six hours—nearly as rapid as the plague. It attacks single women after delivery, as well as the married. Dr. John Clarke was of opinion, that a succession of warm damp seasons and mild winters would induce the disease. This is also the opinion in Dublin (Lawder de Febre Puerperal. Edin. 1821.) ; yet why does not the disease prevail in private life, which is not always the case, if the constitution of the season be the cause of it ?

During the Autumn of 1819, fifty-six women died of puerperal fever at Vienna ; the patients were mostly between twenty and thirty, were of a full habit of body, and three only were emaciated. Seven patients had the disease in the Hospice de Perfectionnement at Paris, during the Autumn of 1824, and all of them were under thirty years of age. (Rev. Med. Janvier, 1827.) Three of these patients recovered by the use of mercurial frictions, the rest died ; and in all the uterus was inflamed, and gangrenous in some parts. Velpeau, Bourgon, and Roux, tried the mercury, as recommended by the surgeon at the Hospital at Antwerp. I shall detail their opinions in this most formidable disease, as soon as I shall have described the report of the Vienna Professors of 1819. These eminent men were appointed as commissioners by the government to inquire into the cause of the epidemic ; and report that 'the fever was preceded by marked changes in the whole system, and particularly in the uterus, indicating clearly an inflammatory state. The lochia disappeared immediately, or in a few hours. The breasts were found empty of milk, loose and flabby. The mouth of the womb was found gangrenous, when the inflammation was most recent. In the last stage of the disease, symptoms of effusion into the chest and abdomen, extreme debility of the powers of life, and gradual dissolution, were apparent. Effusions of a brownish serum, mixed with coagulable lymph, were found in these cavities. The rapid putrefaction of the body in a few hours after death—the dissolved state of the blood, the strikingly soft state of the whole bowels, heart, lungs, liver, spleen, kidneys, and particularly of the womb, indicated a colliquative putrescent condition of the whole system induced by disease. The uterus was gangrenous in every instance, especially at its orifice, and it appeared to spread from this point as from a focus. This was seen in all the bodies, fifty-six in number, all the patients having died, from the 26th of July to the 31st of August. The *treatment* of the disease had from the first been antiphlogistic ; blood-letting, leeches to the abdomen, with emollient cataplasms and clysters, blisters, calomel, and emetics were used. On the commencement of the debility, they had recourse to diffusible stimulants, but with the same unfortunate results. Most of the women experienced, previous to admission, a burning heat in the abdomen, near the pubes ; slight shiverings and distressing pains in the shoulders ; and the greater number had erythematic spots on one of the hands and feet, and chiefly on the joints. Forty of them had laboured under privations before delivery.' During the last puerperal epidemic fever of Edinburgh, 'the disease spared neither high nor low, rich nor poor, those delivered in the Hospitals, in the city, nor in the environs, neither the gay and dissipated, nor the most retired and domestic.' (Edin. Med. and Surg. Journ. July 1824.)—'But,' continue the editors of the last work, 'shall not disease, which in every individual seized with it terminates in gangrene, and in the destruction of the whole organization, which appears as highly malignant, we may say, as pestilential fever, not also at least generate contagious matter, since it is universally allowed, that gangrenous and putrid fever very readily produce contagious matter ? Has not the disease a striking analogy to hospital gangrene, typhus, and even the exotic pestilential diseases ?' For a more detailed account of the subject, I must refer the reader to the Edinburgh Med. and Surg. Journ., and to the Med. Chirur. Review, 1825, vol. ii. The German Reporters advise a separation of the patient from the healthy, though no contagious property could be traced ; a recommendation made by Clarke of Dublin, Clarke of London, Young, and Hamilton of Edinburgh, and Velpeau, Bourgon, and Roux, of Paris. The three last named practitioners, in their report above alluded to, had tried the various modes of treatment recommended by Denman, Leake, Gordon, Hey, Butter, Manning, Walshe, Hulme, Clarkes, Hull, Hamilton, Armstrong, Brenan, Sutton, and Broussais, and all without success.

From fifty to two hundred leeches were applied to the abdomen, as recommended by Broussais and Labatt, with all other means 'to boot,' in fifty cases ; and without success. Even blood-letting from the arm had been freely tried at the same time. The reporters then had recourse to mercury, and ordered inunctions on the abdomen, two drachms of the ointment every two hours, which saved three patients out of seven ; the disease being advanced and hopeless in the fatal cases. Two grains of calomel were given every two hours, at the same time. They think themselves warranted to draw the following conclusions from the facts they observed :—

1st. That puerperal peritonitis once completely established, and abandoned to itself, is almost invariably mortal.

2d. That it remains yet to be proved that sanguineous depletion *alone* is adequate to the cure of the disease.

3d. That the writings of Hamilton, Gordon and Vendenzande, incontestibly prove that, by means of calomel in large doses, many cases of puerperal peritonitis of the most severe kind have been saved.

4th. That mercurial frictions over the abdomen, made at short intervals, promise considerable success, and that this ought to draw the attention of the profession to the said point of practice.

5th. That, by means of mercurial frictions, patients have been rescued, as it were, from the brink of the grave; and consequently that this remedy should be tried, however late in the disease.

6th. That the friction should be continued without fear, until the mouth becomes affected—and in most cases, for some time after all the symptoms have subsided.

7th. That it would probably be advantageous to conjoin the internal administration of calomel, warm baths, and warm temperature.

8th. That the facts observed by the authors, without being sufficiently numerous or conclusive, are yet such as may encourage, authorise, nay, compel practitioners to prosecute the enquiry.

From much consideration on all the opinions on this subject, I think it is obvious that the disease under consideration is a peculiar one, and widely different from common inflammation of the peritoneum, that it commences in the uterus, and is best relieved, perhaps cured by the free use of mercury. Dr. Blundell recommends copious blood-letting as early as possible, and repeated two or three times; but this has failed with the majority of domestic and foreign practitioners. It is rather surprising, that local bleeding has not been more generally employed. Thus leeching and cupping might be applied to the groins, hypogastric region, and the former to the labia and vagina. Again, why not apply cold to these parts, and also to the vagina and uterus. Cold injection, or pounded ice might be introduced into the vagina, and applied to the orifice of the uterus; to that part which is first affected. The only objection to the use of cold would be the presence of the lochia; but this should not prevail, for the application of cold, both externally and internally to the uterine region, would lessen vascular action, increase uterine contraction, and so far from suppressing the lochial discharge, most probably increase it. General bleeding should precede the use of mercury, which will ensure its effects, as on all other occasions.

I conceive great improvement might be made in the application of the mercury. It ought to be given in large doses, as recommended by Boyle in syphilis; by Cartwright in the same disease, in fever, liver complaint, and acute and chronic dysentery (Medico Chir. Rev. 1826, p. 339;) and by Musgrave, in the fevers of the West-Indies. (Edin. Med. and Surg. Journal, 1827, v. 28.)—Musgrave's plan is most applicable to the rapidly fatal disease under consideration, which is the exhibition of calomel, every three or four hours in the following doses, until some effect is produced. He divides two scruples of calomel and one scruple of camphor into twelve papers, and he has exhibited from three to five hundred grains of the former, in fevers of the West-Indies, with success. He informs us, that the camphor promotes the mercurial action.

Again, the mercurial ointment should not only be applied to the abdomen, as recommended by the French, but also to the axillæ, a mode that will often suddenly produce its effects.

Before concluding this article, I should mention the use of the oil of turpentine, as recommended by Dr. Brenan of Dublin. That gentleman published an Essay on the use of this medicine in puerperal fever; and records six cases, four of which it completely cured, and relieved the others. The work, from which I quote, was published in 1814. The four successful cases occurred in the Dublin Lying-in Hospital, and were witnessed by the assistant-physician of that period. The cases occurred in 1812, when the disease destroyed a great number in the hospital. The patients on whom it was tried, were despaired of when the turpentine was exhibited. The quantity of the remedy did not exceed two ounces in any case, and was given in the proportion of half an ounce, or a table-spoonful. It was applied to the surface of the abdomen, and with the effect of relieving the tension of that part. The patients declared themselves suddenly relieved by the medicine. It was exhibited by Hamilton in a few cases, but without success. Blundell and Copland also tried it in this country, but without producing any marked benefit. They tried it, however, only in a few cases. The profession in Dublin also have ceased to employ it. If an ounce or two of oil of turpentine could cure the real puerperal fever, the discovery would justly entitle its author to the especial notice of the government, and to the thanks of posterity. The talented author has not explained the mode of operation of the remedy, nor has he given that minute history of the symptoms of the cases which he has related, that is necessary to establish them as real instances of the disease under consideration. It does not follow, that as puerperal fever ravaged the hospital at the period, every woman, 'who vomited green and yellow bile, and had pain and tension in the abdomen,' laboured under the disease; for it has happened in the same hospital, that a woman lying between the beds of two others, labouring under the disease, was not affected. This is asserted by Lawder and Raymond, in their inaugural dissertations on the disease published at Edinburgh, 1821. The remedy, however, has not been tried fairly by the profession, for I have been unable to find a single practitioner who has employed it in ten cases. All who used it assert it does no injury, and therefore it should be tried with the other remedies, as already described.

Blundell has known the disease to prove fatal in twenty-four hours; and Joseph Clarke was of opinion, it might set in before delivery; a fact that was proved in the German epidemic. Haighton described a case that occurred ten or twelve days after delivery, and proved fatal. It may commence insidiously, and has been mistaken for ephemera, milk fever, after-pains, colic, spasm of the stomach, bowels, and uterus; in fact, most of these diseases are styled puerperal fever, by the majority of practitioners.

In milk fever, the abdomen is soft, and not pained on pressure, the rigors are slight, and the pulse seldom exceeds ninety. The countenance is natural, the breasts are swelled, hot, and tender, and all these symptoms disappear in twenty-four or thirty-six hours.

It can be distinguished from after-pains, they being always periodical, and rather relieved by pressure, and generally removed in twenty hours, the countenance is natural, the pulse is only quick during the severity of the pains, and the lochial discharge is natural; but soon fetid in puerperal fever. Again, in colic, spasm of the stomach, duodenum, and uterus, the pains are periodical, and the pulse scarcely affected, except during the fit. Blundell asserts that the disease may be cured, if the patient be bled within six hours of the chill; and that she will usually die of the disease, if continued for twenty-four hours. The efficacy of bleeding is not confirmed by the reports of the French and German physicians, nor by the statements of many British practitioners. Blundell recommended blood-letting from twenty-five to thirty-five ounces, if the pulse range from one hundred and twenty to one hundred and sixty, and there be pain on pressing the abdomen; the operation to be repeated from four to eight hours if the symptoms be unchanged. If the pulse is lowered, and the pain less, then the operation should not be repeated. He is rather inclined to recommend the bleeding a third time in six hours after the second, if the pulse and tenderness are as urgent as before, 'though from the use of venesection, he fears much benefit is not to be expected. Beware of bleeding, if collapse has begun, or if the two first bleedings have exceeded fifty ounces or more.' An average quantity for a third bleeding is from ten to twelve ounces. The rapidity of the pulse alone, or the tenderness of the abdomen, will not justify the second or third bleeding. The bleeding should be performed in the first twenty-four hours after the chill, and not exceed fifty ounces. Opium, to the amount of ten grains, may be given after the bleedings, watching the effects,

Calomel was given to the amount of two scruples after the first bleeding, and the mouth was soon affected, but the patient died. Blisters are objectionable in the worst forms of the disease, as they render it impossible to decide whether the tenderness be relieved. Leeches in great numbers are objectionable after the second bleeding: the oil of turpentine, as recommended by Brenan, is an excellent rubefacient, and may be applied to the abdomen, until redness is produced. There is great danger from too much depletion, collapse may be hastened by it. Doulcet of Paris, in 1782, recommended ipecacuan emetics, which are now never tried. The pressure caused by the abdominal muscles during vomiting would aggravate the disease. For a full account of the pathology and treatment of this disease, from the earliest period to the present, I refer to my observations in the Lond. Med. and Surgical Journal.

Tonnellé places most reliance on mercurial frictions, which saved three patients. Dr. Isaac A. Johnson has published six cases, in each of which he exhibited half an ounce of turpentine and the same quantity of castor oil every hour, until the bowels were freely purged, and then continued the medicine at longer intervals, and all terminated successfully. (Philadelphia Med. Journ.)—Dr. Payne, of Nottingham, most strongly recommends the oil of turpentine, and has always found it successful. (Edinb. Med. and Surg. 1822, vol. xviii. p. 538.) He never used depletion of late years. In a subsequent essay he informs us that the disease was epidemic in the Winter of 1822. 'Not any of those who were ill of the disease, to whom I was called, were bled, either generally or locally, and every one of them recovered. Augmenting the intestinal secretion, appeared very speedily to put a stop to the complaint in every instance.' (Op. Cit. 1824, vol. xxii. p. 59.) Dr. Dewees, the eminent American obstetric writer, has lately recorded a case of real puerperal fever which was observed by two other practitioners. The antiphlogistic plan was strenuously pursued, but without success. The case was considered hopeless, when thirty minims of oil of turpentine were exhibited every hour, sinapisms applied to the legs, and an ounce of mercurial ointment rubbed on the abdomen every night, with an enema containing one drachm of laudanum. This practice was continued for three successive days—the patient recovered. (Amer. Journ. of Med. Sciences, Aug. 1828.") 650.

A very minute and extensive index is added to the work, and must greatly enhance its value as a manual of reference at the bed-side of the patient. The book may be carried in the pocket of the practitioner, and the varied sources of information which it points out, will render it very acceptable to the lecturer and writer on obstetric subjects.

